# A mixed-methods study investigating the acceptability of an early acceptance and commitment therapy (ACT) intervention to aid adjustment to appearance changes after burns

**DOI:** 10.1016/j.bodyim.2026.102049

**Published:** 2026-03

**Authors:** Laura Shepherd, Fuschia Sirois, Diana Harcourt, Paul Norman, Lance M. McCracken, Andrew R. Thompson

**Affiliations:** a Nottingham University Hospitals NHS Trust, UK; b Durham University, UK; c University of the West of England, Bristol, UK; d University of Sheffield, UK; e Uppsala University, Sweden; f Cardiff University, UK

**Keywords:** Acute burn injury, Appearance, Visible difference, Acceptance and commitment therapy, ACT, Psychological therapy

## Abstract

Appearance concerns after burns start soon after injury. However, early psychological interventions to support individuals with their changed appearance are absent. This study investigated the acceptability of an early acceptance and commitment therapy (ACT) intervention delivered by a psychological therapist either in person or virtually to help individuals adjust to changes to their appearance after burns. As a secondary objective, the potential effectiveness of ProACTive was also investigated. Using mixed-methods, 13 adults (eight women, five men) participated. Acceptability was measured by uptake rates, module completion rates and interview data. Over two-thirds of participants completed all modules. Three main themes were identified from interviews: *An acceptable intervention; Valuing the therapeutic relationship;* and *Early support is key.* Data suggested that ProACTive may be acceptable, although some inconsistencies within the data were observed. Ratings of helpfulness were positive and qualitative feedback suggested that ProACTive seemed helpful by providing space to explore and reflect on appearance changes, developing psychological flexibility and self-compassion, and preparing individuals for being around other people. Positive and negative affect significantly decreased (moderate effect sizes) but no significant changes on appearance concerns, psychological flexibility or self-compassion (small effect sizes) were revealed. ProACTive may be an acceptable early psychological intervention to support the adjustment of appearance changes after burns. Acceptability may be higher in individuals with appearance concerns and those admitted to hospital. The intervention holds promise soon after burns but further research on acceptability and effectiveness is needed.

## Highlights

• ProACTive may be an acceptable early intervention after burns.
• Ratings of helpfulness and qualitative feedback were positive.
• ACT for appearance concerns in acute hospital settings needs further evaluation.

## 1 Introduction

Burns cause sudden changes to the appearance of the skin. These include acute wounds (that typically heal over time) and longer-term or permanent scarring, pigmentation changes or limb loss, creating distress for some individuals (Attoe & Pounds-Cornish, 2015). Individuals can struggle with adjusting to appearance changes, and some will experience distress (e.g., anxiety, self-consciousness, shame) which can lead to impairment in work, social and relational functioning if these persist and entail significant distress (Rumsey and Harcourt, 2012, Sarwer et al., 2022). In a systematic review identifying 33 studies with a range of participants with regard to gender and age, most commonly in North America, findings related to the association between appearance concerns and objective characteristics of burn injuries have been reported as mixed (Cleary et al., 2020). This is consistent with the wider visible difference literature that argues that it is the subjective perception of the severity of an appearance change and psychological variables that are more useful in determining distress related to appearance concerns rather than objective measures of severity (e.g., Moss, 2005).

There is strong evidence that appearance concerns start early following burns. Shepherd et al. (2024a) found that in 15 adults (9 women and 6 men) cared for in British burns services, appearance concerns began during or shortly after hospital admission when individuals see their injuries for the first time and experience a fear of rejection and stigma as a result. Gullick et al. (2014) found that in 9 adults (7 men and 2 women) admitted to Australian burns services, grief for their pre-burn appearance was apparent. Dunpath et al. (2015) also described the influence of a fear of stigma on appearance concerns in 5 adult men cared for in South African burns services. Similarly, Bergamasco et al. (2002) detailed the impact of shame and stigma on relationships with others in 35 adults (23 male and 12 female) cared for in a South American burns service. Acceptable and effective early psychological interventions to support individuals soon after burns and possibly prevent or reduce longer-term difficulties are warranted.

### 1.1 Psychological interventions for appearance concerns

Very few studies have investigated psychological interventions focused on appearance concerns at any stage after traumatic injury such as burns. Research on interventions is typically generic rather than focused on appearance concerns specifically and lack standardised measurements of appearance concerns or control groups (Blakeney et al., 2005, Cooper and Burnside, 1996, Hwang et al., 2019, Seehausen et al., 2015, Shepherd et al., 2020). To date, only one study has evaluated an intervention after burns that specifically measured and focused on appearance concerns, as detailed below (Shepherd et al., 2020). Specifically, acceptance and commitment therapy (ACT: Hayes et al., 1999) was delivered to 3 adults with appearance concerns (2 men with burns and 1 woman with necrotising fasciitis, 5–13 months after injury/onset) who had received care in a British burns service in an uncontrolled case series (Shepherd et al., 2020). The only study that included a control group reported a positive impact of a social skills training programme for 64 adolescents (26 male and 38 female) who sustained burns at least 2 years prior and cared for in a North American burns service (Blakeney et al., 2005).

To date, only one study has considered early psychological intervention for appearance concerns after burns (Cukor et al., 2015). This 14-session intervention (delivered to 10 adults (4 men and 6 women), 1–7 months post-burn) in a North American burns service covered a range of psychological difficulties, with only 3 sessions specifically focussed on appearance. The majority reported improvements in body image and community integration, but Cukor et al. (2015) acknowledged that improving coping with scarring was problematic. The sessions focused on reducing appearance concerns involved social skills training, rather than psychological techniques to respond to distress, and the study lacked a control group. In summary, there is a dearth of research exploring early psychological interventions explicitly focused on appearance concerns after burns.

Systematic reviews have noted there are few high-quality studies evaluating psychological interventions for appearance concerns in adults with any injury or medical condition or treatment that affects appearance (Bessell and Moss, 2007, Muftin and Thompson, 2013, Norman and Moss, 2015). Most of the studies in these reviews have used cognitive behaviour therapy (Clarke et al., 2014), to identify and modify unhelpful thoughts or behaviour, and social skills training, to teach people how to manage other people’s reactions to their appearance, with interventions including individual and group formats, in person and telephone delivery and self-help. In addition, a manual to guide CBT for appearance concerns was developed based on findings from a large multi-centre set of studies (Clarke et al., 2014). However, there is a lack of high-quality evidence for CBT for appearance concerns.

### 1.2 Acceptance and Commitment Therapy (ACT)

ACT is a third-wave psychological therapy incorporating acceptance and mindfulness. ACT aims to enhance psychological flexibility, an overarching process associated with psychological wellbeing. Psychological flexibility involves being open to experiencing distressing internal experiences (e.g., thoughts and emotions), being present-focused, and acting in line with personal values in the presence of distressing experiences (Harris, 2009, Hayes et al., 1999). It can include developing skills for effectively responding to distressing emotions and thoughts or beliefs about the self or others and engaging in behaviour that is meaningful to the individual, all of which are relevant to those with appearance concerns (Rumsey and Harcourt, 2012, Sarwer et al., 2022). In contrast, psychological inflexibility is associated with psychological difficulties and inherently includes experiential avoidance (attempts to control or avoid distressing internal experiences; Hayes et al., 1999).

Self-compassion involves engaging in self-kindness rather than self-criticism, seeing one’s own suffering as common to the human experience rather than feeling isolated and having a balanced perspective on painful thoughts and emotions rather than overidentifying with them (Neff, 2003). Theoretical evidence suggests psychological flexibility and self-compassion are related (Neff and Tirch, 2013, Tirch et al., 2014, Yadavaia et al., 2014). Neff and Tirch (2013) argued that the ability to perspective-take (self-as-context) allows a more detached relationship to inner experiences and cognitive defusion from painful stories. In support of this, Boland et al. (2021) found that experimentally increasing perspective-taking (interpersonally (‘self’ vs. ‘other’) and temporally (‘now’ vs. ‘then’)) reduced emotional discomfort and cognitive fusion and increased self-compassion in a group of 61 (48 female) individuals in the UK. Yadavaia et al. (2014) further argued that both psychological flexibility and self-compassion emphasise mindfulness, and involve an awareness that the self and others have moment-to-moment perspectives, promoting self-as-context and self-compassion.

In a content analysis of the first 1000 randomised controlled trials conducted, ACT was reported to be effective across a wide range of psychological difficulties and physical health conditions to help individuals respond to their distressing internal experiences in ways that help them live meaningful lives (Hayes & King, 2024). Brief ACT interventions have also shown to be effective in physical health populations in a systematic review and meta-analysis (Dochat et al., 2021). Techniques to foster self-compassion can be integrated to further enhance ACT’s ability to help individuals acknowledge and allow distressing inner experiences whilst engaging in valued action (Bernstein, 2025).

A growing body of evidence suggests that difficulties with psychological flexibility are associated with appearance concerns. In a cross-sectional questionnaire study, Shepherd et al. (2019) initially revealed that lower acceptance, cognitive defusion, mindfulness and committed action was associated with increased appearance concerns in 78 individuals (31 male and 47 female) with burns and cared for in a British burns service. Also conducted within British burns services, Shepherd et al. (2024a) detailed a qualitative study describing how 15 adults (6 male and 9 female) after burns had difficulties responding to their early appearance concerns with psychological flexibility due to a fear of rejection and stigma and internalised societal ideals around appearance and gender, and a prospective cohort study also revealed that lower psychological flexibility measured during hospital admission predicted increased appearance concerns two- and six-months later in 175 people (117 male and 58 female) after burns (Shepherd et al., 2025). The role of psychological flexibility in appearance concerns has also been reported in 114 women with breast cancer in Italy (Policardo et al. (2024), 105 individuals (46 male and 59 female) with dermatological conditions treated in a UK hospital (Vasiliou et al., 2023), 121 military veterans (113 male and 8 female) and 197 civilians (119 male and 77 female) with visible injuries in the UK (Keeling et al., 2025), and 220 adults (48 male and 172 female) with a variety of visible differences living in the UK (Zucchelli et al., 2020b). This research provides a theoretical rationale for the use of ACT to support individuals with appearance concerns, including after burns.

Similarly, self-compassion has also been implicated in appearance concerns after burns when studied in addition to psychological flexibility (Shepherd et al., 2024a, Shepherd et al., 2025) and visibly-injured military veterans and civilians (Keeling et al., 2025), as detailed above. The role of self-compassion in appearance concerns in other medical conditions has also been examined, such as in 10 individuals (3 male, 6 female and 1 non-binary) with skin conditions living in the UK (Clarke et al., 2020) and 195 women with breast cancer in Australia (Todorov et al., 2019). A lack of self-compassion was also reported as a barrier to engaging in early psychological interventions for appearance concerns in 15 adults (6 male and 9 female) after burns in the UK (Shepherd et al., 2024b).

Emerging research highlights the promise of ACT as a psychological intervention for appearance concerns. Zucchelli et al. (2020a) reported that ACT delivered in a British service helped 6 women with visible differences caused by injuries and medical conditions live their lives in ways that were more values-consistent, develop ways to influence other people’s reactions and respond to their internal distress and foster self-compassion. As detailed above, Shepherd et al. (2020) described a case series where ACT reduced functional impairment and increased values-based action in three individuals with appearance concerns due to burns and necrotising fasciitis. Siemann et al. (2023) presented a single case description of a Dutch woman with facial palsy with appearance concerns who had positive outcomes after a group intervention based on CBT and ACT. Hotton et al. (2022) evaluated information and self-help therapy guides based on CBT, ACT and social skills training in 88 people (5 male and 83 female) with facial palsy, some of which were focused on appearance concerns and managing other people’s reactions to visible differences, and reported reduced appearance concerns and good levels of acceptability. Powell et al. (2023) conducted a pilot randomised feasibility and acceptability study of a four-week ACT digital self-help intervention in 145 individuals (11 male and 134 female) with a visible difference in the UK. A dropout rate of 68 % was reported, although many rated the intervention as useful (77 %) and helpful (74 %). Powell et al. (2023) suggested that some individuals might have needed a therapist to deliver the intervention. Similarly, Zucchelli et al. (2021) developed a mobile phone app to deliver ACT to people with visible differences in the UK but feedback from 6 user representatives (2 male and 4 female) and 8 clinicians suggested that the technology should not be a substitute for therapist delivered interventions. Despite this promise, the evidence to date is limited to the application of ACT for chronic appearance concerns rather than as an early intervention when appearance concerns are emerging. Self-compassion may also be a therapeutic target for those with appearance concerns using ACT interventions.

### 1.3 Acceptability of early psychological interventions

Acceptability, feasibility, uncertainties and real-world implementation are essential aspects of intervention development and evaluation (Skivington et al., 2021). Acceptability is typically assessed by objective measures of behaviour (i.e., dropout, withdrawal or discontinuation rates) and questionnaires or interviews measuring satisfaction, attitudes and experiences. The Theoretical Framework of Acceptability (TFA; Sekhon et al., 2017) proposes that acceptability is a multi-faceted construct that reflects the extent to which individuals delivering or receiving an intervention consider it to be appropriate, based on cognitive and emotional responses to the intervention that are either anticipated or experienced. It has seven components measuring affective attitude, burden, perceived effectiveness, ethicality, intervention coherence, opportunity costs and self-efficacy.

The only acceptability study of early psychological interventions for appearance concerns after burns (Shepherd et al., 2024b) interviewed 15 individuals (6 male and 9 female) within three months of their injuries in the UK. Early psychological interventions for appearance concerns were perceived as absent from routine burn care but likely to be acceptable if delivered within a therapeutic relationship. However, psychological (e.g., negative beliefs about talking therapies or accepting help/stigma) and contextual (e.g., time restrictions and travel requirements to return to the hospital) obstacles to engagement were identified, similar to obstacles to engaging in support for any psychological difficulty at any stage following burns when this was explored in 11 (5 male and 6 female) individuals who had been treated in a British burns service (McDermott et al., 2023). Shepherd et al. (2024b) concluded that interventions should be embedded into the burn care pathway to enhance acceptability through normalisation and that the optimal time to introduce an early psychological intervention was during hospital admission or the early stage of outpatient care. In summary, early psychological interventions for appearance concerns may be acceptable but further research is needed.

Cukor et al.’s (2015) pilot study detailing a group intervention after burns, as detailed above, evaluates an early psychological intervention for other issues within 7 months post-injury. Cukor et al.’s (2015) intervention focused on improving post-traumatic stress disorder, depression and quality of life, but appearance concerns were also targeted and was deemed to be acceptable and helpful for some individuals. Therefore, it is possible that early interventions for appearance concerns might be delivered alongside or within interventions for other areas of distress after burns. Psychological techniques to aid pain management during dressing changes in the acute period after a burn have also been investigated (e.g., Fauerbach et al., 2002, Haythornthwaite et al., 2001), with mixed findings, suggesting that early interventions more generally shortly after a burn may be acceptable but further research is required.

### 1.4 Aims

The primary aim of the current study was to investigate the acceptability of ProACTive, an early ACT intervention to help individuals adjust to changes to their appearance after burn injuries. As a secondary objective, the study aimed to gather preliminary data on the potential clinical effectiveness of the intervention based on changes on measures of appearance concerns, positive and negative affect, psychological flexibility and self-compassion.

## 2 Method

### 2.1 Design

A mixed-methods acceptability study was conducted. Qualitative data from interviews following the ProACTive intervention were analysed using template analysis, a form of thematic analysis that allows both inductive and deductive reasoning to be applied within the analysis of acquired data (King & Brooks, 2017). Template analysis allows for the development of a priori themes to enable initial coding of interview transcripts, whilst also allowing for novel constructs to be identified as the analysis progresses. Standardised self-report questionnaires were also completed to provide preliminary indications of potential clinical effectiveness.

Adults (18 years or over) with an acute burn injury were invited to participate. Participants started the ProACTive intervention either during their hospital admission to a regional burns unit in the United Kingdom, or within one month of hospital discharge or within one month of their injury if they had received outpatient burn care only. Individuals were excluded if they were: in hospital for reconstructive surgery; too physically unwell to participate; in a mental health crisis (being admitted to hospital with injuries due to self-harm or suicidal behaviour, ongoing concern by the clinical care team about suicidal risk, or challenging behaviour as a consequence of a mental health problem); not fluent in English; known to have a cognitive impairment; or were already receiving psychological support.

Participants were not required to have appearance concerns or distress related to their appearance changes in an attempt to investigate the acceptability of an intervention that could be offered to all burns patients in order to normalise it and embed it into routine burn care (Shepherd et al., 2024b). Therefore, participants could experience a degree of appearance concern or distress or have no concerns about their appearance changes. Furthermore, there were two levels of participation. Firstly, individuals could complete the ProACTive intervention. Secondly, if individuals did not want to complete the ProACTive intervention, they could participate by providing a reason why this was the case. This secondary level of participation supported further learning about the acceptability of the intervention (see Supplementary Materials).

The study protocol was uploaded to the Open Science Framework prior to recruitment (https://doi.org/10.17605/OSF.IO/9DBU5). Ethical approval was obtained from the London - Bromley NHS Research Ethics Committee (REC) and Health Research Authority (IRAS ID: 339105; REC ref.: 24/LO/0402). The study was also registered on ClinicalTrials.gov (identifier: NCT06377709).

### 2.2 Participants

Thirteen adults (eight female, five male) aged between 20 and 78 years (median: 46 years) complete at least one module of the ProACTive intervention. Table 1 displays their pseudonyms and demographic information.

**Table 1:** Pseudonyms and Demographic Information of Participants Completing the ProACTive Intervention.

Pseudonym	BESAA-A mean score	Gender	Age (years)	Ethnic group
Julie	0.9	Female	51	White British
Aadila	1.0	Female	20	Pakistani
Rachel	1.2	Female	62	White British
Csilla	1.6	Female	47	Other White background
Sarah	2.0	Female	40	White British
Robert	2.2	Male	22	White British
Paul	2.4	Male	55	White British
Rose	2.4	Female	78	White British
Hannah	2.5	Female	42	White British
David	2.6	Male	62	White British
John	2.7	Male	46	White British
Elena	2.8	Female	22	Other White background
Neil	3.3	Male	38	White British

*Note.* BESAA-A: Appearance Subscale of the Body Esteem for Adolescents and Adults. Mean scores range from 0 to 4.

Lower scores on the Appearance Subscale of the Body Esteem for Adolescents and Adults (BESAA-A; Mendelson et al., 2001) represent lower body esteem and therefore increased appearance concerns. Scores on this measure at the start of the intervention ranged from 0.9 to 3.3 (median: 2.4), and the measure is further detailed below. Burn injury information was gathered from medical records. Eight participants began the ProACTive intervention during their hospital admission, two started after hospital discharge and three received outpatient burn care only. Time from the burn injury to completing the first module of the ProACTive intervention ranged from 2 to 131 days (median: 8 days). Eight participants began the intervention within 10 days of their injury and eleven participants began the intervention within three weeks of their injury.

Injuries included flame (*n* = 7) and scald (*n* = 6) burns. Total body surface area (% TBSA) burnt ranged from 0.25 % to 60 % (median: 3 %). Depth of burns included partial thickness (*n* = 5), full thickness (*n* = 4), and mixed depth (*n* = 4). Body location of burns included the face/head/neck (*n* = 4), chest/abdomen (*n* = 6), arms (*n* = 5), legs (*n* = 5), hands (*n* = 3), back (*n* = 2), feet (*n* = 2) and genitals (*n* = 1).

One participant received a single session of psychological support from the usual clinical care team during the intervention for an issue unrelated to appearance concerns. No other participants received any burns-related psychological support during the intervention.

### 2.3 ProACTive intervention

The ProACTive intervention consisted of up to five 30-minute sessions (modules), delivered in person or virtually. All intervention modules were delivered by author LS, a Consultant Clinical Psychologist with significant experience in delivering psychological care to individuals following burn injuries and training/supervised clinical practice in ACT, who followed the ProACTive psychological therapist guide, and audio-recorded sessions for fidelity checking. The intervention was based on ACT and aimed to provide early support to individuals in relation to changes to their appearance following burns. It aimed to increase psychological flexibility and self-compassion. It also included social skills training, incorporating ACT concepts. The intervention and associated patient resources were developed by authors LS, AT and LM. Contributions were also provided by a Patient Advisory Group, a group of clinical psychologists working in burn services, and authors DH, PN and FS. As detailed earlier, the intervention was developed so it could be completed whether or not any appearance concerns were present so individuals could use it to help them respond to current distress or any distress they may have experienced afterwards in relation to appearance changes following their burns.

The content of the intervention, including the exercises, scripts and resources, was guided by a review of relevant protocols by author LS. Participants complete the first introductory module but could subsequently choose how many other, and which, modules they wished to complete and the spacing of modules. Patient resources, including scripts of exercises completed during the module and accompanying QR codes/website links to audio-recordings of the exercises on YouTube, were provided following completion of each module. A summary of the intervention is provided in Table 2, and the intervention protocol and patient resources to accompany the intervention are available from the first author upon request.

**Table 2:** Summary of ProACTive intervention.

Module	Aims	Content	Timings
Introduction	Providing an introduction to the intervention; building therapeutic rapport and gaining a sense of the patient’s experiences; validating and normalising the patient’s experiences; self-compassion; developing creative hopelessness; introducing acceptance and contact with the present moment	Introduce the aim of the intervention, acknowledging that it may be useful now, in the future or both	5 min
		Invite the patient to briefly describe their experiences related to how their burn looks and their appearance change(s) and how they are responding to these, incorporating self-compassionate perspective-taking	10 min
		Reflecting on the workability of the patient’s strategies	5 min
		Unwanted party guest metaphor – introducing the idea of reducing the control agenda (acceptance)	5 min
		Mindful breathing exercise (or five senses exercise if breathing creates discomfort)	5 min
Responding to our feelings and thoughts	Promoting acceptance; continuing being present moment focused; self-as-context; self-compassion	Reminding the patient about the unwanted party guest metaphor and reducing the control agenda	5 min
		Passengers on the bus exercise	10 min
		Experiential acceptance exercise (breathing into/making room for/allowing internal experiences)	5 min
		‘Who is noticing?’ reflection, and self-compassionate perspective-taking	5 min
		Mindful breathing exercise (or five senses exercise if breathing creates discomfort)	5 min
Getting distance from our thoughts	Developing cognitive defusion; continuing being present moment focused; self-as-context; self-compassion	Reminding the patient about the unwanted party guest metaphor and reducing the control agenda	5 min
		Experiential cognitive defusion exercise (noticing the thought, naming the story, thanking your mind)	10 min
		Leaves on a stream exercise	5 min
		‘Who is noticing?’ reflection, and self-compassionate perspective-taking	5 min
		Mindful breathing exercise (or five senses exercise if breathing creates discomfort)	5 min
Doing what is important to us	Increasing committed action; acceptance; continuing being present moment focused; self-compassion	Reminding the patient about the unwanted party guest metaphor and reducing the control agenda	5 min
		Values reflection	5 min
		Doing what Matters exercise	10 min
		‘Two sides of a coin’ metaphor	5 min
		Mindful breathing exercise (or five senses exercise if breathing creates discomfort)	5 min
Being around other people	Developing self-efficacy and skills in being around other people and other people’s reactions, utilising acceptance and self-compassion	Allow-Assert-Act with Kindness exercise	20 min
		Taking control when around other people	5 min
		Mindful breathing exercise (or five senses exercise if breathing creates discomfort)	5 min

### 2.4 Data collection

#### 2.4.1 Demographic and burn injury information

Demographic (age, gender, ethnicity) and burn-injury information (mechanism of burn, percentage body surface area burnt (% TBSA)) was gathered for all participants, whether they were completing or had declined the intervention. This information was collected from the medical records by author LS .

#### 2.4.2 Investigating acceptability

##### 2.4.2.1 Descriptive data

Uptake rate, reasons for declining the intervention and details related to module completion were gathered. Modality preferences for receiving post-session resources (in person; post; email) and the number of views of the online YouTube videos containing audio-exercises was also collected.

##### 2.4.2.2 Questionnaires

###### 2.4.2.2.1 Helpful Aspects of Therapy Form (Llewelyn et al., 1988)

This is a seven-item standardised self-report questionnaire that gathers data about helpful and hindering aspects of therapy sessions. It was completed immediately after every module completed by the participant. Participants are asked to rate how helpful the most helpful or important event within the module (and if applicable, a second helpful event) was on a visual analogue scale from 1 (Extremely hindering) to 9 (Extremely helpful). Similarly, they are asked whether anything happened during the module that was hindering, and if so, how hindering this was on a scale from 1 (Extremely hindering) to 4 (Slightly hindering). Participants are also prompted to describe what was most helpful or hindering.

###### 2.4.2.2.2 Questionnaires to assess reasons for ending the ProACTive intervention before completion or declining the intervention

In participants who agreed to complete the ProACTive intervention, a single-item multiple choice question was asked to capture the reason(s) for ending the ProACTive intervention prior to all modules being completed, if applicable (e.g., ‘Now does not feel like the right time for me,’ ‘Practically, it feels too difficult to access sessions’). A similar questionnaire was used for individuals who did not want to complete the ProACTive intervention but consented to providing a reason why. Both questionnaires can be found in the Supplementary Materials.

##### 2.4.2.3 Interview schedule

A priori themes based on template analysis guided the semi-structured interview schedule (King & Brooks, 2017). A Patient Advisory Group comprising individuals with lived experience of burn injuries contributed to the design of the interview schedule. The a priori themes and interview schedule can be found in the Supplementary Materials. The interview schedule was guided by the Theoretical Framework of Acceptability (TFA: Sekhon et al., 2017) and included questions around each component of this model. Questions also explored participants’ views around the flexibility (e.g., ‘What did you think about the intervention being flexible in terms of choosing whatever sessions you wanted, and when?’), delivery (e.g., ‘How important was the presence of a psychologist in the delivery of the intervention to you?’) and timing (e.g., ‘How do you feel about the timing of the intervention, that is was introduced to you whilst you were still in hospital?’) of the intervention. Interviews ranged from 10 to 42 min in duration.

#### 2.4.3 Evaluating potential clinical effectiveness

To gather preliminary data on the potential clinical effectiveness of the ProACTive intervention, the following standardised self-report measures were used.

##### 2.4.3.1 Body Esteem Scale for Adolescents and Adults – Appearance subscale (BESAA-A; Mendelson et al., 2001)

This is a standardised 10-item subscale of a self-report questionnaire that measures appearance concerns/body esteem. The total mean score of this measure was calculated, which has a possible range of values between 0 and 4, with lower scores indicating reduced body-esteem (higher appearance concerns). It has been used in previous research with individuals with burn injuries with an alpha coefficient of.95 reported (Lawrence et al., 2006, Shepherd et al., 2025). In the current study, the internal consistency for the BESAA-A was α = .78 before the intervention and α = .78 after the intervention.

##### 2.4.3.2 Positive and Negative Affect Schedule (PANAS-GEN; Watson et al., 1988)

This is a 20-item standardised measure of positive and negative affect (10 items for each). Higher scores for each subscale represent greater positive or negative affect. It has been shown to have internal consistency of.86–.90 for positive affect and.84–.87 for negative affect, and test-retest reliability of.79 for positive affect and.81 for negative affect over one week (Watson et al., 1988). In the current study, the internal consistency for the positive affect score before the intervention was α = .79 and for the negative affect score it was α = .80. After the intervention, internal consistency for the positive affect score was α = .69 and α = .88 for the negative affect. Measuring positive and negative affect was included to provide an indication of whether the intervention may have any impact on affect generally, aside from appearance concerns specifically.

##### 2.4.3.3 Comprehensive assessment of acceptance and commitment therapy processes (CompACT; Francis et al., 2016)

This is a standardised 23-item self-report measure of psychological flexibility. It has been used in a previous study that explored an online self-help ACT intervention for people with visible differences (Powell et al., 2023). Higher total scores with a possible range of 0–138 were calculated, with higher scores representing greater psychological flexibility. The authors report adequate internal consistency (*r* = .34; Francis et al., 2016) and stronger discriminant validity compared to other psychological flexibility measures (Ong et al., 2020). In the current study, the internal consistency for the CompACT was α = .89 before the intervention and α = .86 after the intervention.

##### 2.4.3.4 Self-Compassion Scale - Short Form (SCS-SF; Raes et al., 2011)

This is a standardised 12-item self-report measure of self-compassion. It has been used in previous research exploring self-compassion and body image in populations with a visible difference, including burns (Shepherd et al., 2025, Sherman et al., 2017). The total mean score of this measure was calculated, which has a possible range of values between 1 and 5. Higher scores indicate higher self-compassion. In the current study, the internal consistency for the SCS-SF was α = .83 before the intervention and α = .81 after the intervention.

### 2.5 Procedure

Recruitment took place between June 2024 and January 2025 at a regional burns service in the UK. Potential participants were approached during their hospital admission, within one month of hospital discharge or within one month of their injury if they had received outpatient burn care only if they were deemed to meet inclusion criteria by a psychologist working in the service. Initially, only inpatients were recruited. However, this was extended in December 2024 to include those within one month of hospital discharge or within one month of their injury if they had received outpatient burn care only. This was due to the frequency of inpatients not meeting inclusion criteria and an increasing pressure within the hospital to discharge patients from the ward, leading to short admissions that posed difficulties for recruitment. During recruitment, the number of inpatients and outpatients receiving care within the burns service was recorded alongside the number of those eligible for the study, the number who took part and those who declined participation. This information is detailed in the Supplementary Materials.

Those who declined the intervention but wished to participate in the study by providing a reason why completed the single-item questionnaire to capture this. Individuals who consented to the intervention were asked to complete the four self-report questionnaires on two occasions, as soon as was feasible before and after the intervention.

After completing the pre-intervention measures, participants completed the introductory module of the intervention. After every completed module, participants were given the accompanying patient resources and they completed the Helpful Aspects of Therapy questionnaire. If participants ended the intervention before all modules had been completed, they were asked to complete the single-item questionnaire to gather their reason(s) for this.

After the end of the intervention (either when all modules had been completed or the participant expressed the wish to end the intervention), a semi-structured interview was completed as soon as possible and within two weeks. This was conducted by a different psychologist working in the service, either in person or over a video or telephone call depending on whether the participant was admitted to hospital and participant preference. Interviews were audio-recorded and then transcribed for analysis. Pseudonyms were assigned. Participants were offered a shopping voucher in recognition of their time spent participating in the semi-structured interview.

### 2.6 Fidelity checking

When recruitment stopped, a selection of audio-recordings was randomly selected for fidelity checking by a Clinical Psychologist external to the study and research team and experienced in supporting people following burn injuries and ACT. The audio-recordings were checked against the ProACTive psychological therapist guide by the external psychologist to check that the modules had been delivered consistently with respect to the therapist manual, and a fidelity checklist was completed. One recording for each of the five modules from five different participants was checked. Data describing fidelity to the intervention can be found in Table 3.

**Table 3:** *Fidelity to the intervention protocol*.

	Not at all consistent with protocol	Partially consistent with protocol	Mostly consistent with protocol	Completely consistent with protocol
Fidelity ratings of sessions checked	0 %	0 %	0 %	100 %
Sessions with missing elements of protocol and relevant information	*n* = 0			

### 2.7 Data analysis

Descriptive statistics were conducted to analyse quantitative indices of acceptability. Inspection of histograms, Q-Q plots and boxplots suggested that many of the questionnaire data values were not normally distributed and highly skewed. Wilcoxon tests were used to compare pre- and post-intervention questionnaire scores to provide a preliminary indication of whether appearance concerns and negative affect decreased, and positive affect, psychological flexibility and self-compassion increased. Rank-based effect sizes were calculated by calculating correlation coefficients (*r*) from the Wilcoxon test *Z* values to understand the strength of effects, according to well-known parameters of small (0.1), medium (0.3) and large (0.5) effect sizes (Cohen, 1992). The Statistical Package for the Social Sciences (SPSS) version 17 was used for descriptive and statistical analyses.

Qualitative data from the Helpful Aspects of Therapy questionnaire were analysed by the first author who delivered the intervention using descriptive thematic analysis and followed the six conventional steps: familiarisation with the data; generating initial codes; searching for themes; reviewing potential themes; defining and naming themes; and producing a written account (Braun & Clarke, 2006). Guidance for template analysis (King & Brooks, 2017) was followed to analyse the interview transcripts, which was conducted by the first author who delivered the intervention. Template analysis is not aligned to any particular philosophical position, deferring this to researchers (King & Brooks, 2017). The data were analysed within a critical realist perspective (Bhaskar, 2010) as all authors take this stance in their research. The researchers assumed that participants’ responses during the interviews represented meaningful psychological phenomenon. However, it was acknowledged that knowledge is generated and perceived through lenses of cultural, historical, personal and social experiences and therefore researchers cannot be completely objective during the research process.

Whilst a priori themes were identified, these were used with the awareness that they may not necessarily prove relevant, useful or meaningful and may be refined or discarded. They were used to guide preliminary coding of an initial subset of the data (4 interview transcripts), which led to additional themes and sub-levels being developed into an initial template, using both inductive and deductive reasoning. The next subset of data (another 4 interview transcripts) was applied to this initial template and it was revised accordingly. This was repeated with the final 4 interview transcripts so that all data had been applied to the latest version of the template, with a priori themes being discarded and refined as necessary, following which interpretation of the template began (following guidance by King & Brooks, 2017). The analysis therefore had 7 stages, completed sequentially and iteratively: 1) familiarisation with the data; 2) preliminary coding; 3) clustering; 4) producing an initial template; 5) developing the template; 6) applying the final template; and 7) final interpretation (King & Brooks, 2017). NVivo version 15 was used to assist with qualitative analysis.

### 2.8 Quality control, rigour and reflexivity

Credibility and rigour can be established using a variety of methods, including auditing the analytic process, keeping detailed audit notes and reflexivity (Malterud, 2001, Spencer and Ritchie, 2012). Template analysis is a form of thematic analysis (King & Brooks, 2017), and therefore it was considered important for researchers to reflect and interrogate how their assumptions and expectations impact on their engagement with the data (Braun & Clarke, 2019). Combined with the critical realist stance taken in the current study, reflexivity was important (Braun and Clarke, 2019, King and Brooks, 2017). A reflective diary was maintained throughout the study by the first author and used to inform reflection as to the concepts evident in the data. Transparency was explicit and an audit trail with detailed notes was kept, which also detailed reflexive comments about how the first author’s own position influenced coding choices/template development and interpretation, in line with the process of template analysis (King & Brooks, 2017). An audit checklist was developed prior to the study commencing and completed at the end of the study by the final author. This included interrogation of NVivo documents and codebooks to check that all interviews were sufficiently and systematically coded, template/themes had been defined and redefined, reflections and summaries justified and explained decisions made throughout the analysis, and quotations and interview excerpts mapped onto and suitably reflected themes. To provide rich and detailed accounts, participant quotes and thick description were also utilised (King & Brooks, 2017). The final author also listened to a selection of interview recordings to ensure quality control by checking that the interviews had been conducted in accordance with the interview schedule and the transcripts accurately captured the audio-recordings. In keeping with utilising reflexivity in qualitative research (Olmos-Vega et al., 2023) the research team continually reflected on the relationship between preconceptions and the emerging findings during team meetings and through field notes. This informed the analysis through the consideration of other relevant theories that explained the data.

### 2.9 Positionality statement

All authors are White, cis gender and able-bodied, in their forties, fifties and sixties. None have personal experience of burn injuries. All authors have experience of researching in the area of burn injuries and other populations with visible differences, psychological care provision for people after burn injuries, and psychological flexibility and self-compassion.

The first author was aware of her ongoing clinical role as a Consultant Clinical Psychologist in a burns service. Specifically, she was aware of her previous experience of working clinically with burns patients who experience appearance concerns, including delivering ACT, which is her preferred psychological approach to supporting people with appearance concerns after burn injuries. The final author also had experience of providing clinical services to people who have sustained burn injuries and had previously developed ACT-based interventions for people with visible differences.

All authors were aware of their positionalities, particularly their pre-existing allegiance to cognitive, third-wave and positive psychological theories, throughout the research and collectively engaged in reflexivity to ensure that the qualitative data specifically enabled space for both inductive and deductive conceptualisation. The authors were mindful of the lens and influences they brought to the research process, and explicit conversations were held about their expectations, experiences and influences that may have impacted the analysis and interpretation of the findings.

## 3 Results

### 3.1 Acceptability of the intervention

#### 3.1.1 Descriptive data

**Fig. 1:**
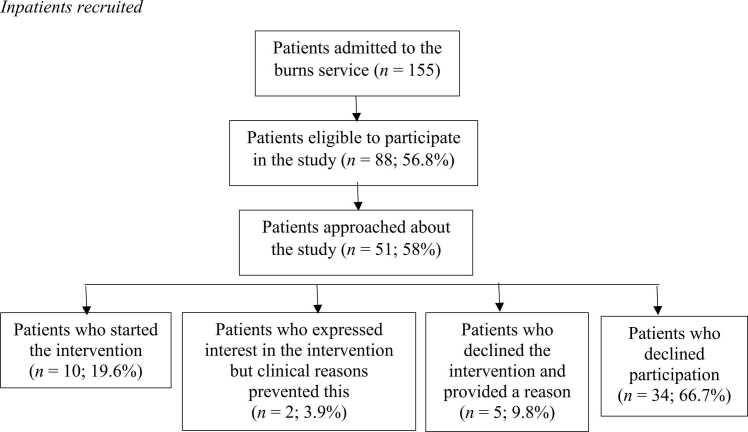
Inpatients recruited.

**Fig. 2:**
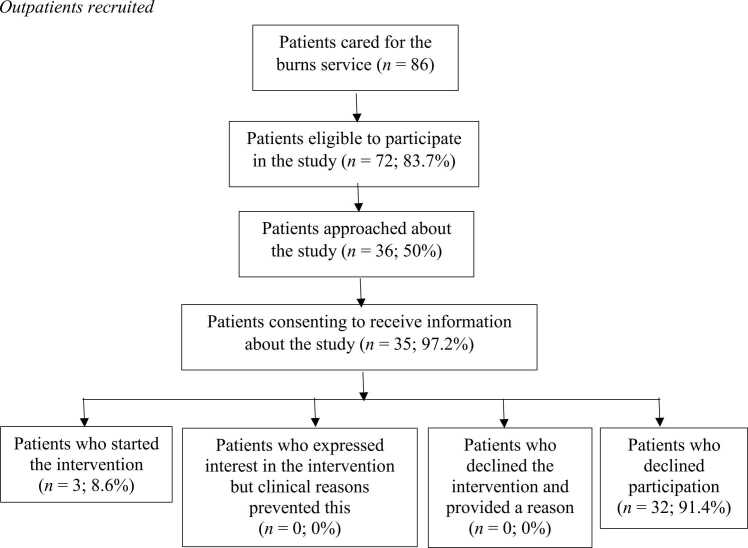
Outpatients recruited.

As presented in Fig. 1, Fig. 2, 58 % of eligible inpatients and 50 % of eligible outpatients were approached about the study. The reason for not approaching the remaining eligible inpatients was pragmatic in that these patients had been discharged from hospital before they were approached by the research team, which had been an initial inclusion criteria before eligibility was widened to include outpatients because this was creating a barrier to recruitment. Furthermore some eligible outpatients were not reached by telephone when contact was attempted.

Of the 51 inpatients and 35 outpatients eligible to participate in the study and were approached and provided with the study information, 19.6 % (n = 10) of inpatients started the intervention and a further 3.9 % (n = 2) expressed interest or intention to start the intervention but clinical reasons prevented this. Therefore, almost a quarter of inpatients expressed interest in the intervention. Of eligible outpatients who consented to being sent the study information, around one in 12 (8.6 %; n = 3) completed the intervention.

Attempts were made to investigate the reasons for declining the intervention in those who were eligible to participate in the study and approached and provided with the study information. Only five (9.8 %) patients, all of whom had been admitted to hospital, who declined the intervention consented to providing a reason for their choice. Three were male and two were female, with a mean age of 51.4 years (range: 20–93 years). All were White British. Mean TBSA was 6.5 % (range: 0.5–20 %). Two participants had flame burns, one had a scald, one received a contact burn, and one had a burn coded as ‘Other.’ Three participants had burns to their chest/abdomen, three had burns on their legs, two had burns on their arms and one had a burn on their back. Participants could select a number of reasons for declining the intervention. Reasons included the intervention not feeling relevant to them (*n* = 3), not being interested in the intervention (*n* = 1), the intervention not feeling like the right time for them (*n* = 1), not having time to participate (*n* = 1), and another non-specified reason (*n* = 1).

Details of module completion are shown in Table 4. Over two thirds (*n* = 9; 69.2 %) completed the entirety of the intervention, one (7.7 %) completed two modules, one (7.7 %) completed three modules and two (15.4 %) completed the introductory module only.

**Table 4:** Number of modules completed, spacing of modules and delivery format.

Pseudonym	No. (%) of modules completed	Days between first and last module	No. of inpatient modules	No. of outpatient modules	Mode of delivery
Rachel	5 (100 %)	10	5	0	In person
John	5 (100 %)	14	0	5	In person
Aadila	5 (100 %)	12	3	2	In person/virtual
Sarah	5 (100 %)	19	1	4	In person/virtual
Robert	5 (100 %)	16	1	4	In person/virtual
David	5 (100 %)	4	4	1	In person/virtual
Csilla	5 (100 %)	8	0	5	Virtual
Elena	5 (100 %)	10	0	5	Virtual
Neil	5 (100 %)	14	0	5	Virtual
Rose	3 (60 %)	10	3	0	In person
Julie	2 (40 %)	4	2	0	In person
Paul	1 (20 %)	-	1	0	In person
Hannah	1 (20 %)	-	1	0	In person

For participants who completed more than the introductory module, remaining modules were completed within four and 19 days (median: 10 days). The main reason for ending the intervention before all modules were completed was practical (*n* = 2; e.g., perceived difficulties accessing the intervention after hospital discharge). For both participants, virtual appointments would have been challenging (one was an elderly woman unable to access video-appointments and one was a woman who preferred in-person appointments. Travel to the hospital was a barrier for both of them). Two participants only completed the introductory module, one of whom reported that appearance changes were not a concern for them. The other participant disengaged from the study and therefore a reason for ending the intervention is not available. Although no formal analysis was completed due to the small sample, differences in module completion did not appear to differ according to gender, ethnicity or burn-related variables.

In line with participant preference, the post-session resources were provided on paper to three (23.1 %) participants and by email to one (7.7 %). Nine (69.2 %) wanted the resources to be provided through a variety of methods (in person, email and/or post).

Between the start of recruitment and one month after the end of data collection, there had only been five views of the online content (audio exercises) on YouTube: Mindful Breathing (*n* = 2); Five Senses (*n* = 2); Allowing Unpleasant Feelings and Thoughts (*n* = 1); and Leaves on a Stream (*n* = 0). It was not possible to identify which participants had accessed these online resources.

#### 3.1.2 Interview data

Twelve participants completed the post-intervention semi-structured interview and one participant was lost to follow-up. Analysis yielded three main themes: *1. An acceptable intervention; 2. Valuing the therapeutic relationship;* and *3. Early support is key.* The full final template can be found in the Supplementary Materials and each theme is detailed below, with supporting quotations. Further quotations are also in the Supplementary Materials.

##### 3.1.2.1 Theme 1: An acceptable intervention

All participants spoke positively about the intervention and the post-session resources. Within this theme, there were seven subthemes that correspond to the components of the TFA (Sekhon et al., 2017): *Affective attitude: Positive views; Burden: Easy and accessible; Self-efficacy: Confidence in using the techniques and resources; Ethicality: It is fair; Opportunity costs: No costs;* and *Perceived effectiveness: A helpful intervention.*

*Affective attitude: Positive views:* This subtheme represents how all participants viewed the intervention in a positive way. All but one participants described liking the flexibility of choosing modules, spacing modules and the delivery format of the intervention (in person/virtually) depending on their needs and preferences. Some also specifically described liking the module structure to provide a clear focus and sense of containment, the length of the modules and how the post-session resources mapped onto the delivered module content. In addition, all but one of the respondents reported enjoying learning skills and techniques to promote adjustment to appearance changes, that they could use now or in the future as described by Sarah:

I know you guys see it all the time but when your face is swollen like that and you're in the first stages you don’t know how long you're going to be like that, but maybe in six months’ time I may, when I've still got a bit of marking, a bit of pigmentation, that may bother me, but I know how to deal with that now, you know, I've worked out how to allow how I'm feeling and deal with it - with the tools.

The value of the post-session resources to use now or in the future to reinforce the skills and techniques was described by some, although others felt that the post-session resources were not necessary over and above the delivered modules. This is consistent with the finding that the online materials on YouTube were minimally accessed, which may suggest that post-session independent practice was not liked as much as the delivered sessions with the psychologist for some participants. However, all respondents described liking the idea of having the post-session resources available to them over time.

*Intervention coherence: A clear intervention aiding reflection and developing skills to respond to appearance concerns:* All but one of the participants believed that the focus of the intervention and how it aimed to work were clear. They understood that the intervention aimed to help people adjust to their changed appearance, reflect on their early reactions and develop skills to respond to these. For example, David explained:

It’s clear to me what I think it aims to do and what it wants to achieve. Um…if the idea of the intervention is to actually challenge or [pause] alter how I felt and what I believed relating to the injury, it’s certainly done that and made think kind of outside my box.

However, two participants (Julie and Rose, who completed two and three modules, respectively, and had reported their reason for ending the intervention was due to difficulty engaging with video-appointments) seemed to struggle with the experience of acceptance underpinning ACT and thus may benefit from additional or more nuanced support to help them reduce their experiential avoidance. For example, Julie described ongoing experiential avoidance, seemingly using the techniques learnt during the intervention as a way to avoid inner experiences as opposed to facilitating openness to experience, highlighting a misunderstanding or difficulty using the techniques within this intended context:

Yeah, well it aims to take your thoughts to a different place, like a happy place, if you’re getting anxious or you said an unwanted guest at a party and stuff and I just said I’d avoid it, you know, I’d try to get rid of the guest, try and get rid of my thoughts.

The other participant was a woman who may have benefited from greater socialisation to the model of psychological flexibility and the aim of ACT, to enhance her understanding of the intervention.

*Burden: Easy and accessible:* All participants typically described the intervention as easy and accessible, placing minimal burden on them. For example, Rachel said, “No effort. It was all very easy, very relaxed. Very logical. I was given time to talk, I was given time to listen, so it was not an effort for me.” The intervention required some cognitive and emotional effort for some people at times. This was largely framed within the aim of the intervention. One respondent talked about burden caused by the physical consequences of the burn, which initially influenced engagement with the post-session resources. No-one else described any burden associated with the post-session resources. However, the above comments related to the post-session resources feeling unnecessary, in addition to the finding that the online YouTube content was not accessed regularly, may suggest that post-session independent practice felt burdensome.

*Self-efficacy: Confidence in using the techniques and resources:* The majority of participants expressed confidence and self-efficacy in using the techniques they had learnt during the delivered modules, and reinforced in the post-session resources, to help them respond to their current and future appearance concerns. For example, Aadila expressed the ability to use the post-session resources: “Because it’s there. Like sometimes if I just need a minute to stop, I’ve got it on my phone if I want to, or if I’m at home I can look at the book.”

*Ethicality: It is fair:* None of the participants expressed any ethical concerns, and all but one of the respondents explicitly described considering the intervention to be ethical, with no concerns around it conflicting with their value systems. This was described by Sarah:

I don’t think it could be – I don’t think there could be an unfair element to it – it’s a tool, it’s there, it’s a service that’s available if somebody feels they need it or, yeah, I don’t think there’s anything unfair about it.

*Opportunity costs: No costs:* All individuals perceived the intervention as having no costs and that no benefits, profits or values had to be given up to engage in the intervention. For example, John said:

No. Again, as soon as I had an appointment I would ring up the person and say “Look, I’ve got this appointment at this time.” I had to travel from [home town] to [location of hospital]. So again, I was more concerned about if trains are late and I can’t make it in time. But no, even when I had, if I arrived early, and one session I arrived early and I called up. And I said “Look, I’m here now. Is there any chance we could do it a bit earlier?” and she was happy to do that. So no, it didn’t really interfere or anything. It fitted in quite well with everything.

*Perceived effectiveness: A helpful intervention:* All participants viewed the intervention as helpful in terms of enabling them to adjust to their appearance changes. Comments related to the intervention feeling helpful were typically around the sessions delivered by the psychologist, rather than the post-session resources or independent practice, although some respondents described using and finding the mindful breathing exercise helpful outside of sessions. Individuals described how the intervention reduced their distress, developed psychological flexibility and their ability to cope with other people’s reactions and improved their ability to do daily activities. Csilla talked about feeling less distressed following the intervention:

All [modules were] effective and all were really helpful and it was really great…it was all good and it was really helpful for me. I feel much better…I don’t have that much deep emotional feelings about it. I mean, I feel better, I feel totally better about it and I can move on.

Increased psychological flexibility, including increased acceptance of inner experiences (e.g., emotions and thoughts), was described by many participants because of the intervention:

The passenger on the bus. That’s it. Yes. That was quite early on. That just made me

realise like, think about what feelings are there and how to deal with them, and they’re always there. Just, you make your own route type thing. So I think it’s a bit of an eye opener. But yeah, it just, it was all right to be fair. It was fine. That was a good one (Neil).

However, the intervention was considered to likely be more useful for those with appearance concerns by a small number of participants who described no or mild appearance concerns and thought that others who were more distressed would find it useful. David reported:

Um…I don’t mean this in a negative way, but it helped but not as much as it would have done had I been concerned about – had I been more concerned about the injury. If I’d been facially scarred then I'm sure the intervention would have been even more helpful but, as it is, with leg scarring and what have you, I'm aware that that can be covered up and hidden away.

##### 3.1.2.2 Theme 2: Valuing the therapeutic relationship

The value and importance of the therapeutic relationship to enable and allow reflection on their inner experiences was described by all individuals. Participants considered the therapeutic relationship to be a key part of the intervention and that without this (e.g., if it was offered in self-help or digital formats), they would not have engaged in the intervention or it would not have been as helpful for them. This is illustrated in the following quotes:

It did impact me. It did make me think, and it did support me, even if it just gave me that half an hour with no-one else around, just me and [psychologist] doing our thing and talking round things. So, it was impactful on the actual day and I'm sure it will impact me as I go forward as well, using what I've been given (Rachel).

With that [self-help or a digital delivery] it just feels very, I think it would feel very, just very fake. Very like ‘oh, it’s just a procedure, you don’t really matter, you’re just a number and we just have to offer it because we have to almost, you know, not because we actually care’, in that way (Elena).

For many, virtual appointments were as acceptable as in-person appointments. However, for a small number, even delivering appointments via video-appointments would have felt less helpful than in-person because of valuing the personal connection with the psychologist. In line with this, as detailed earlier, one participant ended the intervention before they had completed all modules when they were being discharged from hospital due to preferring in-person rather than virtual delivery but travelling back to the hospital for in-person appointments was a barrier.

##### 3.1.2.3 Theme 3: Early support is key

The majority of respondents thought that offering early support to help people adjust to their changed appearance was key, and that the timing of introducing the intervention felt appropriate as it helped people manage their initial emotions and worries about the appearance of the wound and how it might look in the future, as well as the uncertainty around this. Hannah said:

Like I say, I think that’s very important, because I think at that point - for other people particularly, that would be the time when there’s a lot of emotions going off and actually that’s the time when people would need the support to, sort of, try and channel those feelings and channel those and find the help and the support rather than when they’ve left hospital - and it’s almost like the nurse described to me earlier, it’s a safety blanket in here, you know, so to have that sort of support network there before you leave the hospital is a much better idea, in my opinion.

*Getting the timing right when uncertainty around longer-term appearance is present:* In contrast, this subtheme represented the views of a minority of individuals who felt that the intervention might have been better after a bit more time had passed, when the longer-term appearance of the burn was known and uncertainty was removed. These individuals had no or mild appearance concerns, and (like others who felt the timing was appropriate) were not yet sure to what extent their burns would scar and what the scars would look like. Neil reported:

…well I’m still healing, so as I said to you, I’ve not seen the full extent of what it’s going to look like. So like I said to [name of psychologist] at the end, I said maybe something a month down the line, once I’m fully healed, it might be worth doing something like this, because at least then I’ve had chance to look at it, process it and things like that, I think might be the best way for me personally. But I mean other people are different, so –

### 3.2 Potential clinical effectiveness of the intervention

#### 3.2.1 Participant self-report

Data from the Helpful Aspects of Therapy questionnaire suggested that participants rated the intervention as helpful. The median primary rating for each module is presented in Table 5 (on a scale from 1 to 9; Extremely hindering to Extremely helpful, with 5 representing a neutral position). All modules were rated highly in terms of helpfulness and no participants reported any of the modules to be unhelpful, which was consistent with the interview data where all respondents described the intervention as helpful. There was no missing data on this measure.

**Table 5:** *Helpfulness ratings for each module*.

Module	Median rating	Range
Introduction	8	6–9
Responding to our feelings and thoughts Getting distance from our thoughts Doing what is important to us Being around other people	8 8 8 8	5–9 7–9 7–9 7–9

Analysis of the qualitative data from the Helpful Aspects of Therapy questionnaires, where participants described the most helpful or important aspect of the module, identified four main themes: *1. Exploring and reflecting on appearance changes; 2. Developing and reinforcing psychological flexibility; 3. Fostering self-compassion;* and *4. Preparing for being around other people.* These are detailed in turn, with illustrative quotations.

##### 3.2.1.1 Theme 1: Exploring and reflecting on appearance changes

The majority of individuals found it helpful to explore, reflect upon and express their feelings and thoughts related to their changed appearance, facilitated by the intervention. This often enabled a greater understanding of their appearance concerns and prompted reflection about situations that may feel more difficult due to their appearance changes, regardless of the level of distress they reported experiencing. This was described by Csilla who said, “I'm not wearing dresses that show my body, and my wound isn't where others would see it, but it is good to go through thoughts I have, to work out the feelings I have about myself now.” Within this theme, there was a subtheme *‘Valuing the therapeutic relationship.’* This represented several participants valuing the therapeutic space to explore and share distressing experiences related to appearance changes with someone else, which was consistent with the second theme from the interview data which suggested that the therapeutic relationship was key to the acceptability of the intervention. Within this frame, participants found the intervention helpful because they felt listened to and validated. Elena stated:

It was nice to be heard. Giving someone the space to bring a scenario in their heads, with someone else, and not be consumed by it gives a lot of opportunity to think of future problems, insecurities and fears. A problem shared is half-solved.

##### 3.2.1.2 Theme 2: Developing and reinforcing psychological flexibility

All participants described psychological flexibility and how the intervention had developed or consolidated this. For some, it enabled a shift in how inner experiences were viewed, for example, through metaphors. Csilla described how her relationship to her inner experiences had changed in the following quote:

The passengers on the bus exercise was particularly helpful. It's a good exercise because I can do it in the future as well. I can imagine myself as the driver of my bus and I can carry the passengers.

Respondents talked about the value of content or techniques focused on acceptance (e.g., “Learning ways to accept these feelings. The exercise. Realisation of sitting with the feeling” (Sarah)), cognitive defusion (e.g., “The introduction of a completely alien (to me) concept of thanking my mind. Very thought provoking. It made me appraise and think in a completely different direction that I couldn’t have otherwise thought of” (David)) and present moment contact (e.g., “The breathing exercise today. Today has felt really overwhelming and it was a minute to stop. Just feeling relaxed” (Aadila)). A small number also spoke about the usefulness of enhancing self-as-context (e.g., “The observing self. I've never noticed that before. I feel it creates a sense of an observation. You can observe it in a different way and observe different things as well” (Robert)), values identification (e.g., “When I realised my passengers and I had to tell you my values and what is important for me. Because in normal life, you don’t think about these really, and it is good to know” (Csilla)) and committed action (e.g., “Thinking about the challenges to doing something for the first time. It made me think about getting my life back on track” (Rachel)).

##### 3.2.1.3 Theme 3: Fostering self-compassion

Some participants found it helpful to develop an aspect of self-compassion, kindness to oneself, and the ability to respond to internal experiences with self-kindness. Neil described this:

Trying to be kind to myself and think more about myself. You’ve made me think that I don’t think about myself enough, with my feelings and thoughts, and maybe I should think of myself more, instead of being chilled and batting it off all the time.

##### 3.2.1.4 Theme 4: Preparing for being around other people

The intervention also provided a helpful opportunity for several individuals to consider social or relational situations that may feel difficult for them following their burn, reflect on how other people may react to them, and learn skills that may be helpful when around other people. Respondents whose data is represented by this theme had burns to areas of the body visible to other people when clothed (e.g., the head/face/neck, arms or hands). People valued considering specific situations that they were concerned about. For example, Robert described feeling more prepared for social interactions:

Thinking about how people might react and how I might react to them. I can think about what people might say before I see people, and I feel I'd be a lot more prepared for their questions. And also, I'd be less emotional and react better, because I can plan.

None of the participants described any distressing or off-putting elements to the intervention or made any suggestions pertaining to a need for improvement on the Helpful Aspects of Therapy questionnaire. The qualitative feedback on this measure was related to the sessions delivered by the psychologist rather than the post-session resources or independent practice, which is pertinent to note given that the interview data indicated that the post-session resources or independent practice may be comparatively less acceptable.

#### 3.2.2 Changes in questionnaire scores

There was no missing data other than from one participant who was lost to follow-up. Pre- and post-intervention questionnaire scores, the number of participants who improved, worsened and were unchanged on the questionnaires and values from Wilcoxon signed-rank tests are displayed in Table 6.

**Table 6:** Questionnaire scores pre- and post-intervention.

Questionnaire	Median (range) pre-intervention	Median (range) post-intervention	Improved^a^ (*n*)	Worsened^b^ (*n*)	No change (*n*)	Wilcoxon signed- rank values
BESAA-A total mean score	2.3 (0.9–3.3)	2.1 (1.7–3.5)	8	4	0	*Z* = -1.4, *p* = .08
PANAS negative affect subscale score	22 (11−38)	17.5 (11−37)	9	1	2	*Z* = -2.2, *p* = .01**
PANAS positive affect subscale score	25.5 (18−40)	31.5 (24−40)	7	2	3	*Z* = -2.1, *p* = .02*
CompACT total score	82.5 (39−106)	81.5 (59−109)	7	5	0	*Z* = -0.7, *p* = .23
SCS-SF total mean score	2.96 (2.17–4.25)	3.21 (2.08–4.17)	7	3	2	*Z* = -1.2, *p* = .12

*Note.* BESAA-A: Appearance Subscale of the Body Esteem for Adolescents and Adults.

PANAS: Positive and Negative Affect Schedule.

CompACT: Comprehensive assessment of Acceptance and Commitment Therapy processes.

SCS-SF: Self-Compassion Scale - Short Form.

^a^ Improvement reflects increases in BESAA-A, PANAS positive affect, CompACT and SCS-SF scores and decreases in PANAS negative affect scores pre- to post-intervention.

^b^ Worsening reflects decreases in BESAA-A, PANAS positive affect, CompACT and SCS-SF scores and increases in PANAS negative affect scores pre- to post-intervention.

* *p* < .05 level.

** *p* < .01 level.

*p* values are one-tailed.

Table 6 suggests varying outcomes between individuals. On an individual level, two-thirds of participants had decreased appearance concerns (BESAA-A scores) after the intervention compared to before. However, one-third of participants had increased appearance concerns after the intervention. This is not consistent with the qualitative data which suggested that the majority of individuals found it helpful to explore, reflect upon and express their feelings and thoughts related to their changed appearance, and that all participants perceived the intervention as helpful in terms of enabling them to adjust to their appearance changes.

In addition, the number of participants who had increased psychological flexibility (CompACT scores) post-intervention was not too dissimilar to the number who had decreased psychological flexibility post-intervention compared to pre-intervention. This is again inconsistent with the qualitative data where all participants described how the intervention had developed or reinforced their ability to respond to their distress in a more psychologically flexible way.

Furthermore, whereas more individuals had reductions in negative affect and increases in positive affect (PANAS subscale scores) and self-compassion (SCS-SF scores) post-intervention, a minority of individuals showed no or opposing changes on these measures. Considering the qualitative data, only some participants qualitatively described enhancements in self-compassion which seems inconsistent with the questionnaire data on this construct.

At a group level, there were non-significant differences in appearance concerns, psychological flexibility and self-compassion from pre- to post-intervention. However, there was a statistically significant reduction in negative affect and increase in positive affect from pre- to post-intervention.

Considering effect sizes, the statistical analyses revealed a small reduction in appearance concerns (*r* = -0.29), a moderate reduction in negative affect (*r* = -0.46), a moderate increase in positive affect (*r* = -0.44) and small increases in psychological flexibility (*r* = -0.15) and self-compassion (*r* = -0.24). Although effect sizes using *Z* scores from Wilcoxon tests are robust to skewness, severe skew can affect these values and therefore they should be treated with caution.

## 4 Discussion

This study is the first to investigate an early manualised, therapist-led, psychological intervention specifically focused on helping individuals adjust to changes to their appearance after burns. In line with Shepherd et al.’s (2024b) finding that early psychological interventions were likely to be acceptable, the results from this study suggest that the ProACTive intervention may be an acceptable intervention. Firstly, the uptake rate (around a fifth of inpatients and one in 12 outpatients took up the intervention) may be a reasonable indicator of acceptability, particularly for individuals admitted to hospital, given that around a third of adults after burns have been reported to experience appearance concerns (Connell et al., 2013, Shepherd et al., 2017) and the intervention was delivered as part of a research study.

However, a substantial number of those eligible to take part in the study did not. It is difficult to ascertain whether this was a lack of interest/acceptability in participating in the study itself and/or a lack of interest/acceptability in the intervention. Despite attempts being made to seek reasons for declining the intervention to provide some insight into this, only a small number of individuals consented to providing a reason. Reasons included the intervention not feeling relevant to them, not being interested in the intervention, not feeling the right time, and not having the time to participate. The reasons related to the intervention not feeling relevant or of interest is perhaps unsurprising given that more individuals after burns will not experience appearance concerns compared to those who do (Connell et al., 2013, Shepherd et al., 2017). Although, those who provided a reason for declining the intervention were not asked about the presence or absence of appearance concerns, and future research studies would benefit from determining this to particularly identify whether declining the intervention was present in those who were experiencing appearance concerns as this would be important to understand. Furthermore, given the lower uptake rate, acceptability to those discharged from hospital or only receiving outpatient care, may be comparatively lower.

Completer rates were generally high, with over two-thirds of patients completing all five modules, which provides some evidence of acceptability. However, almost a third of participants did not complete the entirety of the intervention. For two participants, this was due to being discharged from hospital and reporting that video-appointments were not acceptable or feasible for them, and that travel to the hospital for in-person appointments was a barrier, although these same respondents also appeared to struggle with the concept of acceptance. One participant described not having any appearance concerns, and a final individual was lost to follow-up. This may further suggest that the intervention is more acceptable to those admitted to hospital, due to the practical barriers that are present in attending appointments following discharge. It may also suggest that the intervention is less acceptable to those who are not experiencing any appearance concerns.

In addition, positive ratings of intervention sessions were given and qualitative feedback from post-intervention interviews suggested that many found the intervention acceptable. The data in the current study mapped well onto the TFA, suggesting that this conceptual framework of acceptability may be useful for evaluating these types of interventions (Sekhon et al., 2017). Themes identified from post-intervention interviews suggest that ProACTive seemed to be liked, perceived as low burden, ethical, coherent, low cost and effective, and appeared to create self-efficacy in practicing skills and techniques taught within the intervention. Interview data also suggested that participants may value the flexibility of the intervention. However, in line with the completer data above, those who had no or mild appearance concerns felt that the intervention would be more useful to people with more significant appearance concerns, suggesting that the intervention may be less acceptable to them.

For some, the intervention appeared to be acceptable whether it was delivered in-person or virtually. However, as detailed above, a small number of participants reported virtual delivery to be unacceptable leading them to end the intervention when they were discharged from hospital, and they also struggled with the concept of acceptance which suggests that they had difficulties with coherence. Similar practical barriers to engaging in psychological interventions generally after discharge from hospital have been described (McDermott et al., 2023, Shepherd et al., 2024b). Moreover, the timing of ProACTive post-burn (typically, within three weeks of injury) seemed to be acceptable for most participants, although a small number queried whether it may be more helpful when more certainty around scarring/longer-term appearance changes was known. This suggests that not everyone felt that the timing of the intervention was acceptable.

There were also some discrepancies between different data sources that are noteworthy and may have implications for acceptability. Firstly, despite respondents’ qualitative feedback, some individuals reported not engaging with the post-session resources or independent practice and the online YouTube materials were only accessed minimally. This may reflect the burden (i.e., time) that this would entail or individuals not liking the idea of practicing the skills independently compared to the sessions delivered by the psychologist. This may be in line with the broader therapy literature which suggests that compliance with out-of-session tasks can be less than 50 % (Bryant et al., 1999). Findings from this study suggest that participants likely did not engage fully with the post-session resources but liked and were reassured by the idea that they were there for the future should they need them. It is therefore not possible to determine how engaged participants were with the intervention outside of sessions they completed with the psychologist (i.e., whether they practiced any of the content independently), and therefore how acceptable the out-of-session aspects of the intervention were. Indeed, the interview data suggests that some individuals may not have felt they needed to practice these outside sessions with the psychologist. It appears likely that the acceptability of the intervention may be higher for the sessions delivered by the psychologist rather than the post-session resources or independent practice. Secondly, all participants described the intervention developing or reinforcing responding to their appearance concerns with psychological flexibility but there was only a small effect size on the questionnaire data pre- to post-intervention.

Although not directly comparable due to employing different methodologies and designs, the acceptability of ProACTive in the current study is consistent with other studies that have found ACT to be an acceptable psychological intervention for chronic appearance concerns after burns (Shepherd et al., 2020) and facial palsy (Siemann et al., 2023) and in women with differing visible differences (Zucchelli et al., 2020a). In the current study, ProACTive had better completer rates compared to Powell et al.’s (2023) self-help, digitally delivered, ACT intervention which had a 68 % drop out rate. Although the findings from the current cannot be directly compared due to it not being a controlled trial, it is possible that a strength of ProACTive in terms of acceptability is that it is delivered by a psychologist and this being of value, as discussed further below.

Individuals appeared to value the therapeutic relationship within ProACTive, and the delivery of the intervention by a psychologist was considered important. Interview data suggested that participants did not think that digitally delivered/self-help formats of the intervention would be acceptable. This is unsurprising given the general importance that is placed on the therapeutic relationship (Rogers, 1959, Wampold, 2015) and is consistent with previous evaluations of digital/self-help interventions for appearance concerns in those with visible differences which has highlighted that digital interventions should not replace in-person interventions (Zucchelli et al., 2021), interventions delivered by therapists are important for at least some individuals (Powell et al., 2023), and that some people struggle to engage in self-help materials for appearance concerns without additional specialist psychological support (Pasterfield et al., 2019). Indeed, the current findings suggested that the post-session resources and independent practice may be less acceptable compared to the sessions delivered by the psychologist, further suggestive of the value placed on the therapeutic relationship that therapist-guided interventions such as ProACTive offers.

In terms of potential clinical effectiveness, there was inconsistency within the data collected in the study. Participants rated the helpfulness of ProACTive highly and no participants described any hindering/unhelpful elements. Qualitative suggested that participants reported that ProACTive reduced their distress, developed their psychological flexibility and self-compassion, prepared them for being around other people and provided an opportunity to explore and reflect on their distress around the appearance of their burns within a valued therapeutic relationship. None of the participants described any distressing or off-putting elements of the intervention or made any suggestions for improvement. However, the questionnaire data found that whilst two-thirds of individuals had improved appearance concerns after the intervention, one-third reported worsened appearance concerns. Differences within the sample as to whether individual questionnaire scores on psychological flexibility, self-compassion (and to a lesser extent, negative and positive affect) changed post-intervention was also notable. With regard to effect sizes, a small reduction in appearance concerns, moderate reduction in negative affect, moderate increase in positive affect and small increases in psychological flexibility and self-compassion were found. Participants had statistically lower negative affect and increased positive affect following the intervention in the current study. However, statistically significant changes in appearance concerns, psychological flexibility and self-compassion were not achieved. Group-level changes in social anxiety and psychological flexibility were similarly not found by Powell et al. (2023).

The current study was not powered to detect statistically significant changes in questionnaire scores. It is not outside the realm of possibility that the intervention might have negative effects on appearance concerns for some individuals. However, there are a number of alternative reasons for the findings and inconsistencies within the data related to this matter, as discussed below.

Firstly, measuring appearance concerns in the early period post-burns may be problematic given that wounds are still healing, their appearance is changing, individuals may not have fully considered how they feel about their appearance changes and some may not yet be aware or have come to terms with the idea that they may be permanently scarred. Indeed, a natural progression for some individuals is for their appearance concerns to increase over time, whereas for others their fears may be allayed or their distress may subside as their wounds heal and they are provided with more information or reassured by the burns team about longer-term appearance changes (Shepherd et al., 2024a). It is possible that, for some individuals, the intervention permitted an exploration of their feelings about their appearance changes or coincided with them making sense of how their wounds may leave lasting scars, through the process of assimilation (Piaget, 1971), leading to worsening scores on the questionnaire measuring appearance concerns despite positive qualitative feedback around the helpfulness of exploring and reflecting on their appearance concerns. If true, this issue could potentially impact the feasibility of any future trials of early psychological interventions for appearance concerns as positive intervention effects could be masked (Norman et al., 1991, Norman and Parker, 1996). It would also indicate that long-term follow-up would be needed to investigate effectiveness. It is also possible given the qualitative data that some individuals may have become more aware of their feelings and thoughts/worries about their appearance changes over the course of the intervention, or have more understanding about their appearance concerns, thereby creating a change in how they responded on the post-intervention questionnaires (Fokemma et al., 2013, Norman and Parker, 1996, Norman et al., 1991).

Furthermore, generic measures of psychological flexibility and self-compassion may not be specific enough to detect changes in appearance-specific psychological flexibility or self-compassion. Context-specific measures of psychological flexibility are generally recommended to detect changes over treatment (Ong et al., 2019) and body-image compassion measures exist (Altman et al., 2017). However, the closest psychological flexibility measures currently available are restricted to those developed for individuals with eating disorders, body dysmorphic disorder or weight concerns which do not map onto the experiences of those with actual appearance changes caused by injuries or medical conditions. Similarly, body-image compassion measures have not yet been used in those with visible differences. As above, it is also possible given the qualitative data that some individuals may have become more aware of their difficulties with psychological flexibility and self-compassion over the course of the intervention, thereby creating a change in how they responded on the post-intervention questionnaires and masking positive intervention effects (Norman et al., 1991, Norman and Parker, 1996).

Finally, it is not possible to determine how engaged participants were with the intervention outside of sessions they completed with the psychologist (i.e., whether they practiced any of the content independently). The interview data suggests that some individuals may not have felt they needed to practice these outside sessions with the psychologist. It is possible that the lack of statistical change on the standardised measures of appearance concerns, psychological flexibility and self-compassion could be due to this and again points to the need for longer follow-up and sample sizes with sufficient power to detect statistically significant changes in future studies. Related to this, it is possible that the statistically significant improvement in positive and negative affect may reflect the common factor of a therapeutic relationship (Rogers, 1959, Wampold, 2015).

### 4.1 Clinical implications

ProACTive may be acceptable to patients with appearance concerns and could be offered to individuals after burns by psychological therapists. However, further research to explore for whom and when this type of intervention would be most appropriate may be beneficial given that there were different views within the sample on these issues. It is also possible that the sequence of modules could influence the acceptability, and future research might also investigate this. It is possible that ProACTive could also be adapted to other populations where appearance has changed due to another type of injury, a medical condition or treatment. The nature, brevity and flexibility of the intervention means that it can be delivered in person or virtually to patients without considerable clinical resource.

ProACTive may be more acceptable to individuals with some degree of appearance concerns. Uptake rates reported in this study suggests that delivering the intervention in routine clinical practice may be feasible. Offering flexibility in delivery (in person or virtually) could be important. The development of digital/self-help formats of ProACTive might not be recommended given the findings. However, it is possible that these alternative formats could be more acceptable at different timeframes after a burn. A small number of individuals will likely require additional support in addition to ProACTive, as the current findings suggest that a small number of participants struggled with acceptance of inner experiences congruent with ACT, instead utilising the techniques to serve the function of experiential avoidance.

It is not possible to determine clinical effectiveness and the possibility that, despite positive qualitative feedback related to helpfulness, some individuals may worsen based on questionnaire scores following the intervention possibly due to the reasons detailed above. It is also not possible to determine whether the trajectories of appearance concerns (and positive and negative affect, psychological flexibility and self-compassion) in those who completed the intervention differed from those who did not, due to the study design. Future research should focus on feasibility of a controlled trial and real-world implications of delivering ProACTive in burns services (Skivington et al., 2021). Training psychologists working in UK burns services in ProACTive has been completed and many have requested the intervention resources so they can consider using them clinically, suggestive of acceptability to psychologists and intended use. However, future research would need to determine whether training is effective, to what extent ProACTive is being used in UK burns services, and psychologists’ experiences of using ProACTive and any barriers or challenges in this.

### 4.2 Limitations

The findings may be subject to social desirability, acquiescence bias or demand characteristics given that the data was collected by the psychologist delivering the intervention (questionnaires) or by a member of the clinical team known to the treating psychologist (interviews). The finding that ProACTive may be acceptable should be interpreted within the context of this limitation. How much the intervention was considered acceptable or useful due to the generic therapeutic relationship rather than the specific ACT content is also impossible to ascertain. Furthermore, it is difficult to establish how engaged individuals were outside of sessions with the psychologist, and therefore how acceptable the post-session resources were. Indeed, some participants reported not using the resources or exercises outside of sessions delivered by the psychologist. Future research should therefore inform how out-of-session engagement can be enhanced and to what extent it is acceptable.

The study cannot make any conclusions about the effectiveness of ProACTive and the study was not powered to investigate this. This should be the focus of future research in addition to the feasibility of controlled trials. The current study initially aimed to recruit inpatients only but experienced recruitment difficulties due to short inpatient admissions. This, and the possible challenges associated with outcome measures, should be considered in future study designs, with respect to feasibility of recruitment. It may also limit the generalisability of the findings around acceptability given that a significant proportion of patients were not offered the study because they had already been discharged from hospital, in line with the original study protocol. Moreover, the study cannot identify what proportion of those with appearance concerns after burns took up the intervention and therefore whether the intervention reached those where interventions are needed the most. In addition, men typically represent almost two thirds of the burn injury population (Mehta et al., 2022, Stylianou et al., 2015) but more women took part in the current study, making it possible that ProACTive is more acceptable to women compared to men and further research is needed to examine this further. Furthermore, a significant proportion of patients were excluded from the study due to not being fluent in English and having complex mental health problems, possibly limiting the generalisability of the findings to these individuals. Further research should investigate the acceptability of ProACTive in these subgroups and in countries outside the UK. Likewise, the intervention in the current study was delivered by a single psychologist and as detailed above, research exploring the acceptability from a practitioner’s perspective would be useful.

Finally, the study cannot make any conclusions about the effectiveness of ProACTive and whether ProACTive can prevent appearance concerns or stop appearance concerns from worsening over time. Future studies aimed at investigating the clinical utility or effectiveness of ProACTive would benefit from a control condition.

### 4.3 Conclusion

ProACTive may be an acceptable early psychological intervention that can be delivered by psychological therapists to support the adjustment of appearance changes after burns. Acceptability may be higher in people with more significant appearance concerns, and in those admitted to hospital due to barriers attending appointments after hospital discharge especially for those who prefer in-person contact. Data related to potential clinical effectiveness was conflicting between qualitative and quantitative indices and, although the study was not powered to detect statistically significant changes, this is important to investigate further. Further research is needed to determine clinical effectiveness and real-world feasibility of delivering ProACTive after burns.

## CRediT authorship contribution statement

**Laura Shepherd:** Writing – review & editing, Writing – original draft, Visualization, Resources, Project administration, Methodology, Investigation, Funding acquisition, Formal analysis, Conceptualization. **Thompson Andrew R:** Writing – review & editing, Visualization, Supervision, Resources, Methodology, Formal analysis, Conceptualization. **McCracken Lance M:** Writing – review & editing, Visualization, Resources. **Diana Harcourt:** Writing – review & editing, Visualization, Supervision, Methodology, Formal analysis, Conceptualization. **Fuschia Sirois:** Writing – review & editing, Visualization, Supervision, Methodology, Conceptualization.

## Funding

Laura Shepherd, Clinical Doctoral Research Fellow, NIHR300282, is funded by Health Education England (HEE) / 10.13039/100006662NIHR for this research project. The views expressed in this publication are those of the author(s) and not necessarily those of the NIHR, NHS or the UK Department of Health and Social Care.

## Declaration of Competing Interest

None to be declared.

## Data Availability

The authors do not have permission to share data.
