# Corrigendum to “A mixed-methods study investigating the acceptability of an early acceptance and commitment therapy (ACT) intervention to aid adjustment to appearance changes after burns” Body Image 56 (2026) 102049

**DOI:** 10.1016/j.bodyim.2026.102080

**Published:** 2026-06

**Authors:** Laura Shepherd, Fuschia Sirois, Diana Harcourt, Paul Norman, Lance M. McCracken, Andrew R. Thompson

**Affiliations:** a Nottingham University Hospitals NHS Trust, UK; b Durham University, UK; c University of the West of England, UK; d University of Sheffield, UK; e Uppsala University, Sweden; f Cardiff University, UK

The authors regret to report that a change is required within the abstract of the article. The abstract details that positive and negative affect significantly decreased after the intervention. The abstract should more specifically detail that positive affect significantly increased and negative affect significantly decreased after the intervention, in line with the findings detailed within the article. The authors would like to apologise for any inconvenience caused.

