# Supplementary material for: A mixed-methods study investigating the acceptability of an early acceptance and commitment therapy (ACT) intervention to aid adjustment to appearance changes after burns
Source: Body Image. 2026 Mar;56:None. doi: 10.1016/j.bodyim.2026.102049 (PMC12961422; doi:10.1016/j.bodyim.2026.102049)
Supplement: Supplementary file 1 — Supplementary material [file mmc1.docx]

**Supplementary Materials**

*Bespoke questionnaires*

Questionnaire

We would really value your honest feedback about your reason(s) for not taking up the intervention, as this will help us to improve and/or make more relevant the support we offer to patients. Please carefully read all answers from the list below before ticking the box(es) to indicate your reason(s) for not taking up the intervention. You may tick as many boxes as you wish, if you think the reason(s) are relevant for you.

I am not interested in the psychological intervention / learning ways to cope with

thoughts and feelings about my changed appearance

I have some concerns/upsetting thoughts and feelings about my changed

appearance, but I do not think the intervention will work/be helpful for me

Now does not feel like the right time for me

I do not think the support is relevant to me

Other (please describe):

_________________________________________________________________________

_________________________________________________________________________

_________________________________________________________________________

_________________________________________________________________________

Thank you for taking part in the study.

Please note that you are welcome to access psychological support through the Burns Clinical Psychology Service at any time in the future, should you wish to access this.

End of Intervention Questionnaire

We would really value your honest feedback about your reason for ending the intervention, if you are willing to tell us. This will help us to improve the support we offer to patients. Please carefully read all answers from the list below before ticking the box(es) to indicate your reason(s) for ending the intervention. You may tick as many boxes as you wish, if you think the reason(s) are relevant for you.

I would prefer not to give a reason

The sessions I have done have been helpful but I don’t think any further sessions will

be of any further benefit to me

I don’t think the sessions I have done so far have been helpful for me

I don’t have time for any further sessions

Now does not feel like the right time for me

Support for changes to my appearance is not my main concern

It feels too difficult to think about my appearance changes at the moment

Practically, it feels too difficult to access sessions (e.g., coming back into hospital for

outpatient appointments, difficulties with video-call appointments)

Other (please describe):

_________________________________________________________________________

_________________________________________________________________________

__________________________________________________________________________________

__________________________________________________________________________________

Thank you.

Please note that you are welcome to access psychological support through the Burns Clinical Psychology Service at any time in the future, should you wish to access this.

*Interview schedule*

| Focus area | Questions |
| --- | --- |
| 1. Introduction | Thank you for taking part in this study. As you know, the aim of the study is to find out if the intervention you received was helpful or not, and how you think it could be improved.  During the interview, I will be asking some questions about this.  Do you have any questions before we start? |
| 2. Affective attitude | How did you feel about the intervention? (Prompts: How much did you like the intervention sessions? Were there sessions you liked more than others, and why? Were there any exercises you liked more than others, and why? How much did you like the resources you were given after sessions to remind you of what was covered?) |
| 3. Burden | How much effort did it take to complete the intervention? (Prompts: What felt the most effortful to you? Were any sessions or exercises particularly burdensome, and why? How much effort and time did it take to engage with the resources you were given after sessions?) |
| 4. Ethicality | Do you think there are any moral or ethical consequences of the intervention, in terms of the content and timing of it? (Prompt: For example, do you think it's fair, or right, to ask patients to do the intervention and why/why not?) |
| 5. Intervention coherence | How clear is it to you how the intervention works or what it aims to do? Please explain. (Prompt: What is less clear about how the intervention works or what it aims to do?) |
| 6. Opportunity costs | Is there anything you missed out on, or could not do, as a result of doing the intervention or engaging with the resources you were given after sessions? (Prompt: For example, did it interfere with any other appointments or care from the burns team, or stop you from doing something else? Please explain) |
| 7. Perceived effectiveness | How much do you think that the intervention was effective in helping you cope with, or adjust to, the changes to your appearance after your burn injury? How were they effective (what impact did they have)?  Which session (or sessions) do you think were the most effective, and why? Which exercise (or exercises) do you think were the most effective, and why?  How effective were the resources you were given after sessions?  What improvements could be made to either the intervention or the resources you were given after sessions, to make it more helpful, and why? |
| 8. Self-efficacy | How confident are you that you can use what you learnt in the intervention to help you cope with changes to your appearance because of your burn injury?  How confident are you about using the resources you were given after sessions, now and in the future? |
| 9. Flexibility | What did you think about the intervention being flexible in terms of choosing whatever sessions you wanted, and when? |
| 10. Delivery | How important was the face-to-face delivery of the intervention to you, by the psychologist?  How would you have felt about a digital delivery of the intervention? (Prompts: For example, if the sessions had been pre-recorded for you to access and listen to/work through on your own? Or, if the intervention could be delivered via an app or electronically somehow that you could complete by yourself, rather than being delivered by a psychologist?)  How did you feel about how the resources were provided to you? Can you suggest any ways to improve how you accessed the resources, or how these were provided to you? |
| 11. Timing | How do you feel about the timing of the intervention, that is was introduced to you whilst you were still in hospital? |
| 12. Close | Thank you so much for taking part in the study.  Do you have any other feedback about the intervention that we have not already talked about? |

*A priori themes*

1. Affective attitude (‘how an individual feels about the intervention’)

2. Burden (‘the perceived amount of effort that is required to participate in the intervention’)

3. Ethicality (‘the extent to which the intervention has good fit with an individual’s value system’)

4. Intervention coherence (‘the extent to which the participant understands the intervention and how it

works’)

5. Opportunity costs (‘the extent to which benefits, profits or values must be given up to engage in the

intervention’)

6. Perceived effectiveness (‘the extent to which the intervention is perceived as likely to achieve its

purpose’)

7. Self-efficacy (‘the participant’s confidence that they can perform the behaviour(s) required to

participate in the intervention’)

8. Flexibility (views around the importance of flexibility within the intervention)

9. Delivery (whether the delivery method/how the intervention was delivered was acceptable)

10. Timing (whether the timing of the intervention was acceptable)

Conceptual definition of acceptability:

‘A multi-faceted construct that reflects the extent to which people delivering or receiving a healthcare intervention consider it to be appropriate, based on anticipated or experienced cognitive and emotional responses to the intervention.’

Reference:

Sekhon, M., Cartwright, M., & Francis, J. J. (2017). Acceptability of healthcare interventions: an

overview of reviews and development of a theoretical framework. *BMC Health Services Research, 17,* 88.

*Final template*

Final template

Study aim: To explore the acceptability of ‘ProACTive,’ an early acceptance and commitment therapy (ACT) intervention, to help burns patients adjust to changes to appearance and prevent distress

1. An acceptable intervention

1.1 Affective attitude: Positive views

1.1.1 Skills building

1.1.2 Flexibility

1.1.3 Structure

1.2 Intervention coherence: A clear intervention aiding reflection and developing skills to respond to appearance concerns

1.3 Burden: Easy and accessible

1.4 Self-efficacy: Confidence in using the techniques and resources

1.5 Ethicality: It is fair

1.6 Opportunity costs: No costs

1.7 Perceived effectiveness: A helpful intervention

1.7.1 Reducing distress

1.7.2 Developing skills to respond to distress with psychological flexibility

1.7.3 Coping with other people’s reactions

1.7.4 More helpful for people with appearance concerns

2. Valuing the therapeutic relationship

3. Early support is key

3.1 Getting the timing right when uncertainty around longer-term appearance is present

*Additional supporting quotations from the semi-structured interviews*

| Theme | Subtheme | Quotations |
| --- | --- | --- |
| Theme 1: An acceptable intervention | Affective attitude: Positive views | “I did move a couple (of modules) about because – the ‘being around people’ I moved ahead because I was going to a work party so obviously being around all my work friends and that since the injury, so I thought it was quite helpful that you could like prioritise things that you were more worried about or meant more to you at the time” (Robert).  “I was looking through the book but I didn’t really go back to anything because I found - at the moment - I found it enough with the time and the sessions I did with [the psychologist]. But it is a good thing because I can go back and have a look again if I feel like it” (Csilla).  “Because you could see what you were hoping to achieve from each bit, so that was really good…Yeah, because they were more like sharp little bite-sized bits” (Hannah). |
|  | Intervention coherence: A clear intervention aiding reflection and developing skills to respond to appearance concerns | “Yeah, well it aims to take your thoughts to a different place, like a happy place, if you’re getting anxious or you said an unwanted guest at a party and stuff and I just said I’d avoid it, you know, I’d try to get rid of the guest, try and get rid of my thoughts” (Sarah). |
|  | Burden: Easy and accessible | “I find it very hard to meditate anyway with the breathing ones and relaxing and trying to tune everything out. But I did like the five senses one where you get to say look at an object, take in the surroundings. I think that was probably one of the best exercises for me, which I got on better with” (John).  “The first couple of days if I’m completely honest, I didn’t read the resources because I was just constantly sleeping. Not until I was actually feeling better did I actually start looking at the resources” (Aadila). |
|  | Self-efficacy: Confidence in using the techniques and resources | “Yeah, I'm very, yeah, I'm really confident, I found it really useful” (Sarah). |
|  | Perceived effectiveness: A helpful intervention | “I can cope with it now, yeah. I’m thinking about it a bit different and I’m not worried. I can cope with it now. I’m not worried about it so much” (Rose).  “Well, I do honestly think that if I hadn’t have had the sessions, I wouldn’t be going out to the shops and stuff” (Julie).  “And it made the way I was feeling more familiar, so it wasn’t – there was less anxiety about it, it was like yeah, yeah, I recognise this feeling, I understand it. I suppose again with being able to allow it was another thing we talked about” (Sarah).  “…it doesn’t particularly help me because I don’t have those feelings. But at the same time I think it should be there because it can help other people” (Elena). |
| Theme 2: Valuing the therapeutic relationship |  | “I think because it’s more of a personal one to one meeting. When you put a screen in the way or a phone in the way, it’s very impersonal and you are talking about something that’s actually happened to you and it is a very personal thing that’s happened to you. So I think to put a screen or a phone in the way of that, it just, to me it’s uncomfortable to do it that way at this point” (Hannah). |
| Theme 3: Early support is key |  | “Good, because I knew at one point I was going to be discharged and you know, obviously my appearance had changed dramatically, so it was like a bit of reassurance that it was done in the hospital and not outside” (Julie). |

*Additional supporting quotations from the Helpful Aspects of Therapy questionnaire*

| Theme | Subtheme | Quotations |
| --- | --- | --- |
| Theme 1: Exploring and reflecting on appearance changes |  | “Asking me how I would feel in the situation I was worried about. It brought my attention to it and what I was anticipating. Just helping me think about different ways to drill down into stuff, if you know what I mean” (Sarah). |
|  | Valuing the therapeutic relationship | “Talking about my thoughts. I always find it helpful to talk to someone who understands” (Rose). |
|  |  |  |
| Theme 2: Developing and reinforcing psychological flexibility |  | “The unwanted guest at the party. I try a lot to block it out and push it away, but it's not always the best way” (Robert). |
| Theme 3: Fostering self-compassion |  | “Introducing the concept of supporting someone else in the same position and then turning that on it's head to support myself. It made me think and put various ideas from this intervention into a context that I was unable to imagine. I feel a dillard - thinking how I could support someone else but turning that towards yourself - I've never thought of that” (David). |
| Theme 4: Preparing for being around other people |  | “Working through a scenario which is coming up - first visit to the café outside of the safe environment of the ward” (Rachel). |
